# Consensus siRNA for inhibition of HCV genotype-4 replication

**DOI:** 10.1186/1743-422X-6-13

**Published:** 2009-01-27

**Authors:** Abdel Rahman N Zekri, Abeer A Bahnassy, Hanaa M Alam El-Din, Hosny M Salama

**Affiliations:** 1 Virology and Immunology Unit, Cancer Biology Department, National Cancer Institute, Cairo University, 1st Kasr El-Aini st, Cairo, Egypt; 2 Pathology Department, National Cancer Institute, Cairo University 1st Kasr El-Aini st, Cairo, Egypt; 3 Tropical Medicine, Faculty of Medicine, Cairo University, Kasr El-Aini st, Cairo, Egypt

## Abstract

**Background:**

HCV is circulating as a heterogeneous group of quasispecies. It has been addressed that siRNA can inhibit HCV replication in-vitro using HCV clone and/or replicon which have only one genotype. The current study was conducted to assess whether siRNA can inhibit different HCV genotypes with many quasispecies and to assess whether consensus siRNA have the same effect as regular siRNA.

**Methods:**

We generated two chemically synthesized consensus siRNAs (Z3 and Z5) which cover most known HCV genotype sequences and quasispecies using Ambium system. Highly positive HCV patient's serum with nine quasispecies was transfected *in-vitro *to Huh-7 cell line which supports HCV genotype-4 replication. siRNA (Z3&Z5) were transfected according to Qiagen Porta-lipid technique and subsequently cultured for eight days. HCV replication was monitored by RT-PCR for detection of plus and minus strands. Real-time PCR was used for quantification of HCV, whereas detection of the viral core protein was performed by western blot.

**Results:**

HCV RNA levels decreased 18-fold (*P *= 0.001) and 25-fold (*P *= 0.0005) in cells transfected with Z3 and Z5, respectively, on Day 2 post transfection and continued for Day 3 by Z3 and Day 7 by Z5. Reduction of core protein expression was reported at Day 2 post Z3 siRNA transfection and at Day 1 post Z5 siRNA, which was persistent for Day 4 for the former and for Day 6 for the latter.

**Conclusion:**

Consensus siRNA could be used as a new molecular target therapy to effectively inhibit HCV replication in the presence of more than one HCV quasispecies.

## Background

Hepatitis C virus (HCV), a member of the *Flaviviridae *family of viruses, is a major cause of chronic hepatitis and hepatocellular carcinoma [1,2]. Viral clearance during acute HCV infection is usually associated with a multispecific CD4^+ ^and CD8^+ ^T cell response, which is weak or undetectable in subjects who do not control the infection [3-5]. Importantly, most chronically infected patients, especially those with genotype 4, fail to resolve HCV infection after combination therapy with pegylated IFN and ribavirin [6-8]. The HCV genome is a positive-stranded 9.6-kb RNA molecule consisting of a single ORF, which is flanked by 5 and 3 UTR. The HCV 5-UTR contains a highly structured internal ribosome entry site [8-13]. The HCV ORF encodes a single polyprotein that is 3,008–3,037 aa in length and is post- translationally modified to produce at least ten different proteins: core, envelope proteins (E1 and E2), p7, and nonstructural proteins (NS2, NS3, NS4A, NS4B, NS5A, and NS5B) [2,13,14]. Despite considerable advances in understanding the function of these proteins, the basic mechanism(s) of HCV replication remains unclear. The recent development of H CV culture and expression of HCV proteins in stably transfected human cells has facilitated the analysis of the role of cellular pathways required for HCV replication and the efficacy of antiviral drugs [15,16].

RNA interference (RNAi), originally discovered in plants, *Caenorhabditis elegans*, and *Drosophila*, is also induced by dsRNA [17,18]. In this process, dsRNA is cleaved into 21–23 nucleotides (known as short interfering RNA or siRNA) by an RNase III-like enzyme known as Dicer [19-21]. These siRNA molecules associate with a multiprotein complex known as the RNA-induced silencing complex and target homologous mRNA for degradation [19-21]. RNAi, which can function independently of IFN-induced pathways, is also effective in mammalian cells [20,22,23]. This suggests that plants and animals share a conserved antiviral mechanism leading to specific destruction of nonself dsRNA [24,25]. RNAi interferes with the replication of a number of animal viruses including HIV-1, flock house virus (FHV), Rous sarcoma virus, dengue virus, and poliovirus [26,27].

As an RNA virus, HCV is a prime candidate for RNAi. Indeed, it has been demonstrated recently that HCV-specific siRNA can inhibit levels of a fusion NS5B-luciferase reporter transcript when the siRNA and the target were cotransfected hydrodynamically into mice [8]. Also, Kapadia et al., [28] demonstrated that by using HCV-specific siRNA, HCV RNA replication and protein expression are efficiently inhibited in Huh-7 cells that stably replicate the HCV replicon (genotype-1) and that this effect is independent of IFN.

In the current study, we compared the effect of consensus siRNA (specific for both the 5-UTR and Core) and regular siRNA in inhibiting ongoing HCV genotype-4 replication using a recently developed HCV-4 tissue culture system in Huh-7 cells.

## Materials and methods

### Design and Synthesis of siRNA

**Figure 1:**
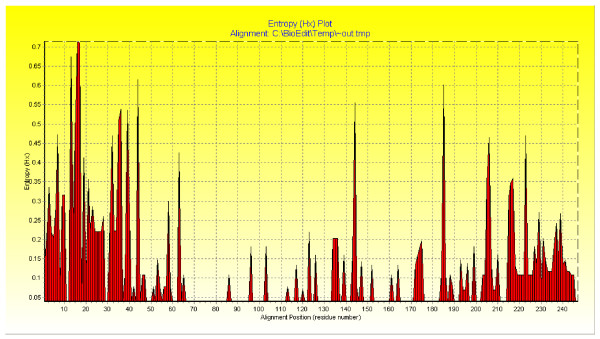
**Position entropy plots for sequences of all patients**.

**Figure 2:**
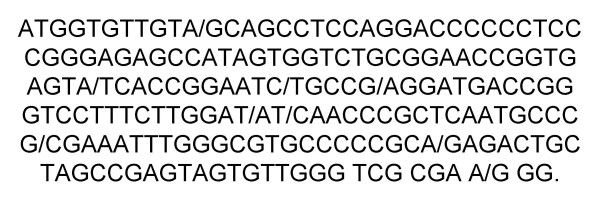
**The generated consensus sequence of the all patients (5'UTR) used for generation of HCV specific siRNA**.

The current study included 60 patients positive for HCV RNA by RT-PCR. Their HCV RNA 5-UTR was previously sequenced in our lab by TRUGENE method [29,30]. Their gene accession numbers were: AY661552, AY673080–AY673111, AY624961–AY624986, AY902780–AY902787. BIOEDIT V 7.0 program [31] was used for sequence alignment editing, visualization, and conservation, and positional entropy plots of all 155 sequences generated (representing all quasispecies from those patients) and were used to generate the consensus sequence (see additional file 1) as shown in Figures 1, 2. This consensus sequence was used as a base for generation of different siRNA using Ambion web-based criteria. Five HCV-specific siRNA were detected based on this consensus of the HCV 5-UTR. However, only two siRNA were selected which showed 100% alignment with HCV sequences in the gene data base and were able to align in both core and 5-UTR. HCV-5UTR-Z5 59 {3'-AACCCGCTCAATGCCCG/CGA-5') 79, sense strand siRNA (3'-CCCGCUCAAUGCCCG/CGATT-5'), antisense strand siRNA: (3'-UCG/CGGGCAUUGAGCGGGTT-5'} and HCV-5UTR-Z3 41{3'-AAATTTGGGCGTGCCCCCGCA-5') 57, Sense strand siRNA: (3'-AUUUGGGCGUGCCCCCGCATT-5'), antisense strand siRNA: UGCGGGGGCACGCCCAAAUTT-5'}. This area has no cross alignment with any other sequences on the gene data base and within IRES of the HCV-5-UTR. These siRNAs were chemically synthesized, HPLC purified and sterilized with ultra-filtration to remove any interfering substances that might be toxic to culture systems.

### Huh-7 cell culture

Human hepatocellular carcinoma cell line Huh-7 was used to establish the *in vitro *HCV replication. Huh-7 culturing and infection were carried out according to previous protocols [32]. Briefly, Huh-7 cells were maintained in 75 cm culture flasks (Greiner bio-one GmbH, Germany) containing Dulbecco's Modified Eagle's Medium (DMEM) supplemented with 4.5 g/L glucose and 10 g/L L-glutamine (Bio Whittaker, a Combrex Company, Belgium), 100 ml/L fetal calf serum (FCS), 10 g/L penicillin/streptomycin and 1 g/L fungizone 250 mg/L (Gibco-BRL life Technologies, Grand Island, NY (USA). The complete culture medium (CCM) was renewed every 3 days, and cells were passaged every 6–10 days. The exact cells count was recorded in 50 μl aliquots after mixing with equal volume of trypan blue (5 g/L; Biochrom KG, Berlin, Germany). A total of 3 × 10^6 ^cells were suspended in 10 ml complete medium and incubated at 37°C in 5% CO_2_.

#### Viral inoculation and sample collection

Viral inoculation and cell culture were done as previously described by el-Awady et al. [33]. Briefly, cells were grown for 48 h to semi-confluence in CCM, washed twice with FCS-free medium, then inoculated with 500 μl serum obtained from HCV infected patients (RT-PCR and antibody positive) (500 μl patient sera and 500 μl FCS-free DMEM/3 × 10^6 ^cells). The HCV genotype in the used sera was previously characterized as genotype-4 with 9 quasispecies based on the method described earlier [34]. The viral load in the used serum was quantified by real time PCR. The average copy number was 580 × 10^6^copies/ml. After 180 min, Ham F12 medium (Bio Whittaker, a Combrex Company, Belgium) containing FCS was added to make the overall serum contents 100 ml/L in a final volume of 10 ml including the volume of human serum used for infection as mentioned above. Cells were maintained overnight at 37°C in 5% CO2. The next day, adherent cells were washed with CCM and incubation was continued in CCM with 100 ml/L FCS. Throughout the culture duration, the viral RNA in Huh-7 cells was assessed qualitatively by sodium dodecylsulphate polyacrylamide gel electrophoresis (SDS-PAGE), for western blotting of viral core antigens. RT-PCR amplification of sense and anitsense strands were tested quantitatively by real time PCR as discussed below.

### siRNA Transfection Protocol Optimization

Since cells vary greatly with respect to their capacity to be transfected, the transfection protocol for each cell line should be determined empirically. Therefore, MAPK1 control Kit (Qiagen GmbH, D-40724 Hiden), GAPDH siRNA and the GAPDH negative control siRNA (Ambion) were used to optimize: 1) optimal cell plating density, 2) optimal type of transfection agent either siPORT amine (Silencer siRNA Transfection Kit Austin TX, USA Cat#1630) or siPORT Lipid (Silencer siRNA Transfection Kit Austin TX, USA Cat# 4505), 3) optimal amount of siPORT transfection agent and whether to transfect in serum-free or serum-containing medium, and 4) optimal amount of siRNA. Accordingly, a highly purified siRNA was obtained from Ambion and transfected using siPORT *Lipid Kit *according to the manufacturer's instructions. Briefly, 0.5–2 × 10^5 ^Huh-7 infected with HCV were plated in 12-well plates and after 3 days 100 nM of siRNA were transfected using siPORT transfection agent. Total RNA was harvested at various times post-transfection using TRIZOL reagent (Life Technologies, Grand Island, NY). Human GAPDH siRNA was used as a control for HCV-siRNA.

### Western blot analysis of HCV core antigens in Huh-7 cells with and without siRNA

Uninfected Huh-7 cell and infected Huh-7 cells with and without siRNA cell lysates were subjected to SDS-PAGE as previously described [32]. After three washes, membranes were incubated with diluted peroxidase-labeled anti-human IgG/IgM antibody mixture at 1:5000 in PBS-3 g/L for previously treated strips with the anti-core (Novocastra, Novocastra Laboratories, UK) for 2 hr at room temperature. Visualization of immune complexes on the nitrocellulose membranes was done by developing the strips with 0.01 mol/L PBS (pH 7.4) containing 40 mg 3,3',5,5 tetramethylebenzedine and 100 μl of 30 ml/L hydrogen peroxide (Immunopure TMB substrate Kit, PIERCE, Rockford, IIIinois, USA)

### PCR of genomic RNA strands of HCV

#### Primers and probe

The primer used for reverse transcription (RT) of HCV RNA was HCV-6: 5'-ACC.TCC-3' (nucleotides [nt] 319 to 324 [29]. The antisense PCR primer for HCV was RB-6B (5'-ACT.CGC.AAG.CAC.CCT.ATC.AGG-3' [nt 292 to 312]) and the sense primer was RB-6A (5'-TG.AGG.AAC.TAC.TGT.CTT.CAC.G-3' [nt 47 to 68]). The oligonucleotide RB-6P (5'-TTG.GGT.CGC.GAA.AGG.CCT.TGT.GGT.ACT.G-3' [nt 264 to 291]) was labeled at the 5' end with digoxigenin and was used as a probe in hybridization experiments to determine the specificities of the PCR products. The HCV oligonucleotides are specific for the 5' un-translated region of the HCV genome. The RT-PCR and the RNA template production were performed as previously described [29,30,32-35]

### Northern Blot Analysis

Total RNA was extracted from all cell types at Days 1, 2, 3, 4, 5, 6, 7 and 8 post transfection, and 5 ug of total RNA were loaded onto the gel. HCV probe was generated from a *Bgl*II fragment (47–1,032 bp) of the HCV plasmid pMOZ-1-HCV using the MAXIscript *In vitro *transcription kit (Ambion). Probing for the GAPDH transcript was performed as described. Both probes were purified using the MicroSpin G-50 columns (Amersham Pharmacia). Blots were visualized and quantified as previously described [35]

### Detection of plus and minus-strand RNA by nested RT-PCR

Detection of plus- and minus- HCV strand was done according to el-Awady et al. [32,33]. The Step One real-time PCR system (Applied Biosystems) was used.

### Quantification of human GAPDH mRNA

We checked the integrity of the cellular RNA preparations from HCV infected Huh-7 cells, by quantification of *GAPDH *mRNA in the absence and presence of siRNA Z3 and siRNA Z5 respectively to ensure that the siRNA used in this study did not adversely affect the expression of a house keeping gene from host cells. *GAPDH *mRNA levels were quantified by real time RT-PCR using TaqMan technology with *GAPDH *specific primers [33]. Amplification of human *GAPDH *transcripts was performed using the TaqMan EZ RT-PCR kit (Applied Biosystems, Foster City, CA). The target template was the purified cellular RNA from Huh-7 cells at 1, 2, 3, 4, 5, 6, 7 and 8 days post infection with HCV, in absence and presence of our siRNA. Reverse transcription-PCR was done using a single-tube, single-enzyme system. The reaction exploits the 5'-nuclease activity of the rTth DNA polymerase to cleave a TaqMan fluorogenic probe that anneals to the cDNA during PCR between the forward primer at nucleotide position 1457 and reverse primer at nucleotide position 3412 of the human *GAPDH *gene. In a 50 μl reaction volume, 1.5 μl of RNA template solution equivalent to total cellular RNA from 2.5 × 10^5 ^cells were mixed with 200 nM forward primer, 100 nM reverse primer, 100 nM *GAPDH *probe, 300 μM from each of dATP, dCTP, dGTP and 600 uM dUTP, 3 mM manganese acetate, 0.5 u rTth DNA polymerase, 0.5 u Amp Erase UNG, 1× Taqman EZ buffer and amplified in the sequence detection system ABI 7700 (Applied Biosystems, Foster City, CA). The RT-PCR thermal protocol was as follows: Initial UNG treatment at 50°C for 2 minutes, RT at 60°C for 30 minutes, deactivation of UNG at 95°C for 5 minutes followed by 40 cycles, each of which consists of denaturation at 94°C for 20 seconds and annealing/extension at 62°C for 1 min.

### Reverse Transcription and Real-Time PCR Analysis for HCV

Total RNA was harvested with Trizol and purified as recommended by the manufacturer (Invitrogen). One microgram of total RNA was incubated with DNase 1 by using the DNA-free kit (Ambion). cDNA was generated by using the TaqMan reverse transcription reagents kit (Applied Biosystems) according to manufacturer recommendations. Reactions with no reverse transcriptase enzyme added were performed in parallel with most experiments and yielded no PCR products. Real-time PCR (Applied Biosystems) was performed. To quantify HCV transcript levels, dilutions of the in-vitro transcribed HCV-plasmid PMOZ-1-HCV plasmids [35] containing the HCV 5-UTR and core or the human GAPDH gene were always run in parallel with cDNA from the Huh-7 for use as standard curves (dilutions ranged from 10^8 ^to 100 copies of each plasmid). The PCR primers for GAPDH are based on the human GAPDH mRNA sequence (GenBank accession no. NMX002046), and spans introns two and three of the GAPDH gene (base pairs 1,457–3,412). The PCR primers for quantitative real-time PCR were HCV RB6A 5'-TGAGGAACTACTGTCTTCACG-3' (sense) and RB6B 5'-ACTCGCAAGCACCCTATCAGG-3' (antisense) [29] and GAPDH 5'-GAAGGTGAAGGTCGGAGTC-3' (sense) and 5-GAAGATGGTGATGGGATTTC-3' (antisense).

### Statistical analysis

The data shown in Figures 5 and 6 were carried out at least in triplicates for each treatment and data averages with standard errors of the means are shown.

## Results

**Figure 3:**
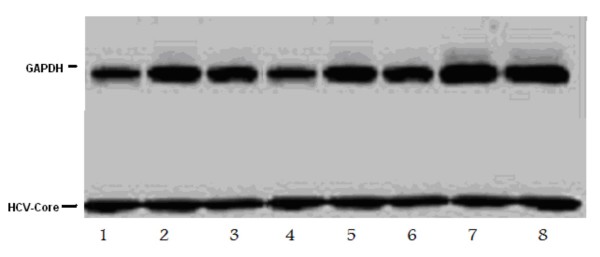
**The expression level of the viral core and GAPDH in Huh-7 cells infected by HCV genotype-4 from day 1 to day 8**.

Since HCV replication in cell culture is limited to Huh-7 cells and their derivatives, we first verified that HCV can replicate in the Huh-7 cells through detection of the viral proteins Core by western blotting as well as detection of viral copies by both real time PCR and b-DNA in both cells and supernatant starting from Day 7 post transfection Figure 3 shows the expression level of the viral core and GAPDH in Huh-7 cells infected by HCV genotype-4 from day 1 to day 7 (Table 1 and Figure 3).

**Table 1:** The reduction rate of HCV-Core protein expression and HCV-RNA level in Huh-7 cells supporting HCV replication inhibited by siRNA.

Days	**RNA titer (untransfected) IU/ml	**RNA titer (Z3 used) IU/ml	**RNA titer (Z5 used) IU/ml	* Core protein reduction rate (Z5 used)	* Core protein reduction rate (Z3 used)
1	4 × 10^6^	4 × 10^6^	3.6 × 10^5^	10%	0%

2	3.9 × 10^6^	3 × 10^6^	2.4 × 10^5^	60%	75%

3	4.1 × 10^6^	> 1000	> 1000	90%	100%

4	4.2 × 10^6^	> 1000	> 1000	100%	100%

5	4.5 × 10^6^	2.2 × 10^6^	> 1000	100%	50%

6	3.8 × 10^6^	2.89 × 10^6^	> 1000	100%	30%

7	3.7 × 10^6^	3.1 × 10^6^	1.6 × 10^6^	40%	30%

8	4.4 × 10^6^	4 × 10^6^	4.2 × 10^6^	0%	0%

* Core protein expression by Western Blot

** RNA titer by Real Time PCR

Next, we assessed whether HCV antigen expression could be silenced by using HCV-specific siRNAs. Two different siRNA (Z3 and Z5) which target both 5-UTR and core region were designed according to genotype 4 consensus sequence (see material and methods). The integrity of this region is associated with optimal translation of the HCV polypeptide, and its sequence is maintained in all HCV sequences in the gene data base. Huh-7 cells containing HCV were transfected with 100 nM of the siRNA and plated for eight days.

**Figure 4:**
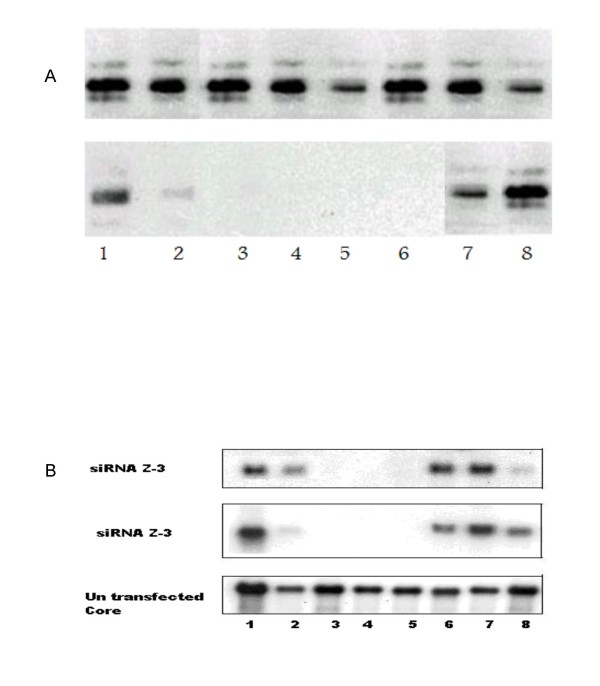
**A. The expression level of the viral core in Huh-7 cells infected by HCV genotype-4 from day 1 to day 8**. Upper row showed HCV-core expression in un-transfected cells. Lower row showed the HCV- core expression in siRNA-Z5 transfected cells. 4B The expression level of the viral core in Huh-7 cells infected by HCV genotype-4 from day 1 to day 8. Upper row showed HCV-core expression in un-transfected cells. Lower row showed the HCV- core expression in siRNA-Z3 transfected cells.

Protein lysates were made and immunoblots were performed with mAbs specific for the HCV core. Viral proteins levels were decreased after 24 hr of the transfection of 100 nM of siRNA specific for HCV, and this decrease continued for seven days with both Z5 and Z3 siRNA (Figure 4A&B). Maximal inhibition of HCV transcript levels was detected on Day 3 post transfection with Z5 and Z3 siRNA and continued for three days by Z3 and six days by Z5 (5.2- and 8.0-fold for Z3 and Z5; respectively). No significant inhibition of HCV transcript levels was detected in cells transfected with the negative control siRNA (*P *= 0.4927; table 1).

**Figure 5:**
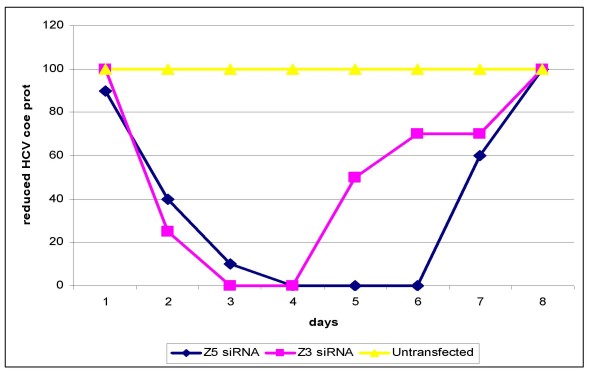
**The reduction in HCV- core protein after transfection with Z5 and Z3 siRNA in Huh-7 cells harboring HCV-genotype-4**.

Total RNAs were harvested from both non-transfected and transfected cells as well as from tissue culture supernatant of both cultures at 1, 2, 3, 4, 5, 6,7 and 8 days after transfection. Quantitative analysis by real time PCR revealed that HCV RNA levels decreased 18-folds (*P *= 0.001) and 25-folds (*P *= 0.0005) in cells transfected with Z3 and Z5; respectively, on Day 2 post transfection and continued for Day 3 by Z3 and Day 7 by Z5 (Fig. 5, 6 & table 1).

**Figure 6:**
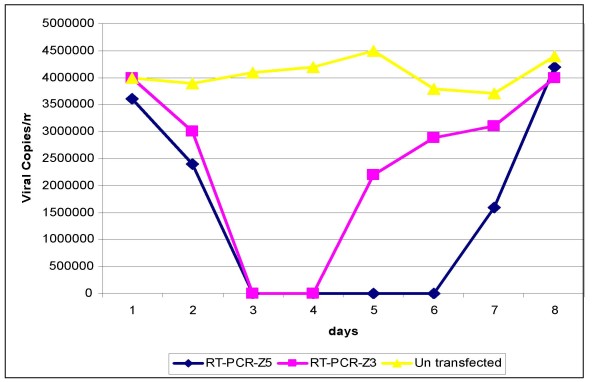
**The reduction in HCV copies/ml after transfection with Z5 and Z3 siRNA in Huh-7 cells harboring HCV-genotype-4**.

## Discussion

In many cases, it is difficult to eradicate HCV infection even with an intensive antiviral therapy that utilizes pegylated interferon-α and ribavirin [25,26]. Although a number of other antiviral compounds, including inhibitors against the NS3-4A protease[36] and NS5B RNA dependent RNA polymerase [37] are currently being tested for their therapeutic applicability; such attempts have not always been promising.

The HCV genome is a positive-sense single-stranded RNA that functions as both a messenger RNA and replication template via a negative-strand intermediate, making it an attractive target for the study of RNA interference. Some studies have demonstrated that siRNAs interfere with HCV gene expression and replication [8,28] others have reported the use of siRNA against HIV-1, HPV and poliovirus in culture cells [27]. In the current study, we were able to show that the introduction of siRNA-5-UTR into target cells that contained HCV, caused a dramatic decrease of viral RNA and protein levels (Figures 4 and 5). This effect was likely due to the degradation of HCV messenger RNA by the RISC endonuclease. We noticed that the effect of RNAi on HCV replication occurs very early after 24 hours post siRNA transfection. Our data is in agreement with McCaffrey et al., [8] who showed that, a fragment of the HCV NS5B RNA polymerase gene, which was transiently co-transfected with siRNA into mouse liver by hydrodynamic injection, was cleaved after treatment with siRNA. Our data are also consistent with those of Randall and associates [38] who demonstrated that siRNA targets and cleaves the HCV 5'UTR efficiently and specifically. More importantly, they showed that the cleavage of HCV-RNA not only suppressed viral protein synthesis, but also blocked the replication of sub-genomic viral RNA.

It is well known that, viruses, particularly RNA viruses such as HCV, are notoriously prone to errors during their replication, and continuously produce mutated viral proteins to escape immune-system defense mechanisms [39]. These mutations may also escape attack by siRNAs. The protein-coding sequence of the HCV genome that was targeted in the study by McCaffrey et al., [8] varies considerably among different HCV genotypes, and even among strains of the same genotype [40]. In addition, given the high error rate of the non-proofreading HCV RNA-dependent RNA polymerase, the so-called 'siRNA escape mutants' which have silent mutations in the protein-coding sequence, could emerge quickly. In contrast, the 5'UTR, which was selected as a target in the present study, is almost identical among the known strains of HCV. Moreover, structural constraints on the 5'UTR, in terms of its ability to direct internal ribosome entry and translation of viral proteins, would not permit escape mutations.

Therefore, the 5'UTR of the HCV genome appears to be an ideal target for siRNA in clinical applications. Not all 5'-UTR-directed siRNAs were equally effective; among the siRNAs tested by Yokota et al. [41], siRNA 331, which is directed against a region upstream of the start codon, was the most efficient, whereas siRNA 82, which is directed against helix II, had almost no effect on viral genome expression. These results may be due to the highly folded structure of the 5'UTR, which may leave few single-stranded gaps that siRNAs can access. Additionally, it has been reported that the target region of siRNA 331 is also an efficient target site for a catalytic RNA, a hammerhead ribozyme, for the suppression of HCV protein expression [42]. Yokota et al., [41] demonstrated that the secondary structure of the HCV RNA genome influences the efficiency of siRNAs at least in part. They also showed that the siRNAs suppressed the expression of an HCV replicon more potently than did the IRES reporter vector. This stronger suppressive effect of siRNA on the HCV replicon might be due to several effects on its autonomous replication mechanism. The blockage of the IRES-mediated synthesis of the nonstructural proteins, which is essential for viral RNA synthesis, and the cleavage of elements in the 5'UTR that are necessary to prime complementary RNA strand synthesis, may result in further suppression of viral replication. They were also able to show that siRNAs not only reduced viral protein synthesis, but also abolished intracellular replication of the viral genomic RNA, raising the possibility that RNAi could achieve the elimination of viruses from persistently infected host cells. Cleavage of the HCV IRES by siRNAs may lead to complicated effects on protein translation. It has been reported that the most 5'part of the UTR may negatively regulate the IRES function [43]. Moreover, deletion of the nucleotides that make up helix 1 leads to an increase in IRES-mediated translation [44]. Yokota et al., [41] suggested that the cleavage of helix I by siRNA 12 led to an enhancement of IRES mediated translation through the inactivation of cis- or trans-acting negative regulatory elements of the IRES.

Our results demonstrate for the first time that careful selection of target sequences for siRNAs is mandatory, not only to achieve maximum efficiency (as with siRNA Z5), but also to avoid adverse effects in therapeutic applications. We elected to make use of Huh-7 cells infected with native viral particles from HCV type-4 positive serum, the most prevalent type in Egypt. We were able to maintain these cells in culture for more than six months. The cells were also capable of supporting HCV replication as indicated by consistent synthesis of plus and minus RNA strands by nested RT-PCR and by real-time PCR technique.

As efficient and safe delivery methods of siRNAs to cells *in vivo *that can suppress HCV replication in all infected cells have not been established yet, chemically modified synthetic siRNA might easily be made and delivered into cells on their own. Recently, it was reported that serum (ribonuclease)-resistant modified siRNA can be delivered into cells without a cationic lipid carrier [45]. On the other hand, the great variability in RNA sequences between different quasispecies and genotypes of HCV makes the use of one siRNA less effective in the therapeutic applications. Therefore, several different combinations of siRNA are necessary to target a particular region of the genome.

To assess this hypothesis we used consensus siRNA which considered four siRNAs at the same time and showed a great inhibitory effect. We showed that the two siRNAs we selected, Z3-siRNA (nt 41–57; from the 5'UTR and nt 173–189 from the core area) and Z5-siRNA- (nt 59–79 from the 5'UTR and nt 109–129 from the core area), completely inhibited viral replication in culture, thus confirming earlier reports on siRNA and suggesting a potential therapeutic value in HCV type-4. Our preliminary data indicate that siRNA Z5 efficiently suppresses HCV replication *in vitro*.

We conclude that the utility of siRNA as a therapy against HCV infection will depend on the development of efficient delivery systems that induces long-lasting RNAi activity. HCV is an attractive target because of its localization in the infected liver, an organ that can be readily targeted by nucleic acid molecules and viral vectors. Also, gene therapy offers another possibility to express siRNAs that target HCV in a patient's liver. The data in this study suggest that siRNAs targeting 5'UTR viral polymerase can elicit an anti-HCV response in cell culture. It represents a promising therapy that could eliminate viral RNA from the infected cell and potentially cure patients with HCV. In conclusion, the efficiency of our siRNAs in inhibiting HCV replication in cells suggests that this RNA-targeting approach might provide an effective therapeutic option for HCV infection, especially at the optimal site within the conserved 5'UTR. Also, double hit Z5 siRNA is more effective in the inhibition of viral replication at the same concentration of Z3 siRNA.

## Abbreviations

siRNA: Silent interfering RNA; RT-PCR: Reverse transcription-polymerase chain reaction; ORF: Open reading frame; 5'UTR: 5' untranslated region; IFN: interferon; FCS: Fetal Calf Serum; GADPH: glyceraldehydes-3-phosphate dehydrogenase; SDS-PAGE: sodium dodecyl sulfate polyacrylamide gel electrophoresis ; PBS: Phoshate buffer saline; NS: Nonstructural; IRES: Internal ribosome entry site.

## Competing interests

The authors declare that they have no competing interests.

## Authors' contributions

ARNZ participated in designing the siRNA, conducted all the practical part of the experiment, entitled the paper, and coordinated the whole work team.

AAB helped in the practical part in the in vitro culture and molecular analysis.

HMAED helped in the practical part of the DNA sequencing part, and helped in editing the manuscript.

HMS the clinician responsible for providing samples for DNA sequencing

## Supplementary Material

Additional file 1 **The alignment of HCV sequences typed by TRGUENE (accession numbers **AY661552, AY673080–AY673111, AY624961–AY624986, AY902780–AY902787**) using CLUSTAL analysis in the Bioedit program**. The data shows an alignment of previously published HCV 5'UTR sequences of all study cases. Click here for file
