# Supplementary material for: Consensus siRNA for inhibition of HCV genotype-4 replication
Source: Virol J. 2009 Jan 27;6:13. doi: 10.1186/1743-422X-6-13 (PMC2661880; doi:10.1186/1743-422X-6-13)
Supplement: Additional file 1 — The alignment of HCV sequences typed by TRGUENE (accession numbers AY661552, AY673080–AY673111, AY624961–AY624986, AY902780–AY902787) using CLUSTAL analysis in the Bioedit program. The data shows an alignment of previously published HCV 5'UTR sequences of all study cases. [file 1743-422X-6-13-S1.rtf]

Figure 1
The alignment of HCV sequences typed by TRGUENE (accession numbers AY661552, AY673080–AY673111, AY624961-AY624986, AY902780-AY902787) using CLUSTAL analysis in the Bioedit program.


             ....|....| ....|....| ....|....| ....|....| ....|....| ....|....| 
                      10         20         30         40         50        60                
AY624965     ---TCTMAGC CAT-VGCGTT A-------GT ATGA-GTGTT -GTACAGCCT -CCAGGACCC 
AY624966     --CGTCT... ...-G..... .-------.. ....-..... -..G...... -......... 
AY624967     ---GTCT.KG ...-R.S... .-------.. ....-..... -..G...... -......... 
AY624968     -AG..AT... ...RG..... .-------.. ....-..... -......... -......... 
AY624969     ---------- -----..... .-------.. ....-..... -......... -......Y.. 
AY624970     ---------- ------.... .-------.. ....-..... -......... -......... 
AY624971     --CGTCT... ...-G-.... .-------.. ....-..... -..G...... -......-.. 
AY624972     AGCGTCT... ...-G..... .-------.. ....-..... -......... -......... 
AY624973     -----CT... ...-GC-... .-------.. ....-..... -......... -......... 
AY624974     GCG...A-.. ...-R..... .-------.. ....-..... -..G...... -......... 
AY624975     ---...A-.. ...-G..... .-------.. ....-..... -......... -......... 
AY624976     --G...AT.. ...-R..... .-------.. ....-..... -......... -.......Y. 
AY624977     CGTCTAW-.. ...-R..... .-------.. ....-..... T......... T......... 
AY624978     --G...A-.. ...-R..... .-------.. ....-..... -......... C.AG.AT... 
AY624979     ---GTYT... ...-GR.... .-------.. ....-..... -......... -......... 
AY624980     --G...A-.. ...-R..... .-------.. ....-..... -......... -......... 
AY624981     -CG...W-.. ...-R..... .-------.. ....-..... -......... -......... 
AY624982     ---------- ---------- -------G.. ....-..... -......... -......... 
AY624983     ---------. ...-G..... .-------.. ....-..... -......... -......... 
AY624984     -CG...A-.. ...-G-.... .-------.. ..-.-..... -......... -......... 
AY624985     --------.. ...-G..... .-------.. ....-..... -..G...... -......... 
AY624986     GCG...A-.. ...-R..... .-------.. ....-..... -......... -......... 
NR7preT      --------.. ...-G..... .-------.. ....-..... -......... -......... 
NR7postT     --------.. ...GG-.... .-------.. ....-..... -......... -......... 
BT1preT1     --------.. ...-G..... .-------.. ....-..... -..G...... -......... 
BT1preT2     --------.. ...-G..... .-------.. ....-..... -..G...... -......... 
BT1postT1    --------.. ...-G..... .-------.. ....-..... -..G...... -......... 
BT1postT2    --------.. ...-G..... .-------.. ....-..... -..G...... -......... 
BT1postT3    --------.. ...-G...A. .-------.. ....-..... -..G...... -......... 
BT1postT4    --------.. ...-G...A. .-------.. ....-..... -..G...... -......... 
BT1postT5    --------.. ...-G...G. .-------.. ....-..... -..G...... -......... 
BT1postT6    --------.. ...-G...G. .-------.. ....-..... -..G...... -......... 
BT2preT1     --------.. ...-G...-- .-------.. G.AG-A.... -......... -......... 
BT2preT2     --------.. ...-G...-- .-------.. G.AG-A.... -......... -......... 
BT2postT1    --------.. ...-G...-- .-------.. G.AG-A.... -......... -......... 
BT2postT2    --------.. ...-G...-- .-------.. G.AG-A.... -......... -......... 
BT2postT3    --------.. ...-G...-- .-------.. G.AG-A.... -......... -......... 
BT2postT4    --------.. ...-G...-- .-------.. G.AG-A.... -......... -......... 
BT3preT1     --------.. ...-G..... G-------.. ....-..... -..G...... -......-.. 
BT3preT2     --------.. ...-G..... G-------.. ....-..... -..G...... -......-.. 
BT3postT1    --------.. ...-G..... G-------.. ....-..... -..G...... -......-.. 
BT3postT2    --------.. ...-G..... G-------.. ....-..... -..G...... -......-.. 
BT3postT3    --------.. ...-G..... G-------.. ....-..... -..G...... -......-.. 
BT3postT4    --------.. ...-G..... G-------.. ....-..... -..G...... -......-.. 
BT3postT5    --------.. ...-G..... G-------.. ....-..... -..G...... -......-.. 
BT3postT6    --------.. ...-G..... G-------.. ....-..... -..G...... -......-.. 
BT3postT7    --------.. ...-G..... G-------.. ....-..... -..G...... -......-.. 
BT3postT8    --------.. ...-G..... G-------.. ....-..... -..G...... -......-.. 
BT4preT1     --------.. ...-G..... .-------.. ....-..... -......... -......... 
BT4postT1    --------.. ...-G..... .-------.. ....-..... -......... -......... 
BT4postT2    --------.. ...-G..... .-------.. ....-..... -......... -......... 
BT4postT3    --------.. ...-G..... .-------.. ....-..... -......... -......... 
BT4postT4    --------.. ...-G..... .-------.. ....-..... -......... -......... 
BT5preT1     --------CT ...-G..... G-------.. ....-..... -......... -......... 
BT5preT2     --------CT ...-G..... .-------.. ....-..... -......... -......... 
BT5postT1    --------CT ...-G..... .-------.. ....-..... -......... -......... 
BT5postT2    --------CT ...-G..... G-------.. ....-..... -......... -......... 
BT5postT3    --------CT ...-G..... .-------.. ....-..... -......... -......... 
BT5postT4    --------CT ...-G..... .-------.. ....-..... -......... -......... 
BT5postT5    --------CT ...-G..... G-------.. ....-..... -......... -......... 
BT5postT6    --------CT ...-G..... G-------.. ....-..... -......... -......... 
BT5postT7    --------CT ...-G..... G-------.. ....-..... -......... -......... 
BT5postT8    --------CT ...-G..... .-------.. ....-..... -......... -......... 
R1preT Q1    --------.. ..G-GCA... .-------.. .A..TA..A. -......... -......... 
R1preT Q2    --------.. ..G-GCA... .-------.. .A..TA..A. -......... -......... 
R1preT Q3    --------.. ..G-GCA... .-------.. .A..TA..A. -......... -......... 
R1preT Q4    --------.. ..G-GCA... .-------.. .A..TA..A. -......... -......... 
R1preT Q5    --------.. ..G-GCA... .-------.. .A..TA.... -......... -......... 
R1preT Q6    --------.. ..G-GCA... .-------.. .A..TA.... -......... -......... 
R1preT Q7    --------.. ..G-GCA... .-------.. .A..TA.... -......... -......... 
R1preT Q8    --------.. ..G-GCA... .-------.. .A..TA.... -......... -......... 
R1preT Q9    --------.. ..G-GCA... .-------.. .A..T...A. -......... -......... 
R1preT Q10   --------.. ..G-GCA... .-------.. .A..T...A. -......... -......... 
R1preT Q11   --------.. ..G-GCA... .-------.. .A..T...A. -......... -......... 
R1preT Q12   --------.. ..G-GCA... .-------.. .A..T...A. -......... -......... 
R1preT Q13   --------.. ..G-GCA... .-------.. .A..T..... -......... -......... 
R1preT Q14   --------.. ..G-GCA... .-------.. .A..T..... -......... -......... 
R1preT Q15   --------.. ..G-GCA... .-------.. .A..T..... -......... -......... 
R1preT Q16   --------.. ..G-GCA... .-------.. .A..T..... -......... -......... 
R1preT Q17   --------.. ..G-GCA... .-------.. ....TA..A. -......... -......... 
R1preT Q18   --------.. ..G-GCA... .-------.. ....TA..A. -......... -......... 
R1preT Q19   --------.. ..G-GCA... .-------.. ....TA..A. -......... -......... 
R1preT Q20   --------.. ..G-GCA... .-------.. ....TA..A. -......... -......... 
R1preT Q21   --------.. ..G-GCA... .-------.. ....TA.... -......... -......... 
R1preT Q22   --------.. ..G-GCA... .-------.. ....TA.... -......... -......... 
R1preT Q23   --------.. ..G-GCA... .-------.. ....TA.... -......... -......... 
R1preT Q24   --------.. ..G-GCA... .-------.. ....TA.... -......... -......... 
R1preT Q25   --------.. ..G-GCA... .-------.. ....T...A. -......... -......... 
R1preT Q26   --------.. ..G-GCA... .-------.. ....T...A. -......... -......... 
R1preT Q27   --------.. ..G-GCA... .-------.. ....T...A. -......... -......... 
R1preT Q28   --------.. ..G-GCA... .-------.. ....T...A. -......... -......... 
R1preT Q29   --------.. ..G-GCA... .-------.. ....T..... -......... -......... 
R1preT Q30   --------.. ..G-GCA... .-------.. ....T..... -......... -......... 
R1preT Q31   --------.. ..G-GCA... .-------.. ....T..... -......... -......... 
R1preT Q32   --------.. ..G-GCA... .-------.. ....T..... -......... -......... 
R3preT Q1    --------.. ...-G..... .-------.. ....-..TG. -..G...... -.....--.. 
R3preT Q2    --------.. ...-G..... .-------.. ....-..TG. -..G...... -.....--.. 
R4preT Q1    --------.. ...-G..... .-------.. ....-..... -......... -......... 
R4preT Q2    --------.. ...-G..... .-------.. ....-..... -......... -......... 
R5preT Q1    --------.. ...-G..... .-------.. ....-..... -......... -......... 
R6preT Q1    --------.. ...-G..... .-------.. ....-..... -......... -......... 
R6preT Q2    --------.. ...-G..... .-------.. ....-..... -......... -......... 
R8preT Q1    --------.. ...-G..... .-------.. ....-..... -..G...... -......-.. 
R8preT Q2    --------.. ...-G..... .-------.. ....-..... -A.G...... -......-.. 
R9preT Q1    --------.. ...-G..... .-------.. ....-..... -......... -......... 
R9preT Q2    --------.. ...-G..... .-------.. ....-..... -......... -......... 
R10preT Q1   --------.. ...-G...A. .AAGGTTA.. ....-....A -..G...... -......T.. 
R10preT Q2   --------.. ...-G...A. .AAGGTTA.. ....-....A -..G...... -......T.. 
R10preT Q3   --------.. ...-G...A. .AAGGTTA.. ....-....A -..G...... -......T.. 
R10preT Q4   --------.. ...-G...A. .ACGGTTA.. ....-....A -..G...... -......T.. 
R10preT Q5   --------.. ...-G...A. .ACGGTTA.. ....-....A -..G...... -......T.. 
R10preT Q6   --------.. ...-G...A. .ACGGTTA.. ....-....A -..G...... -......T.. 
R10preT Q7   --------.. ...-G...A. .AGGGTTA.. ....-....A -..G...... -......T.. 
R10preT Q8   --------.. ...-G...A. .AGGGTTA.. ....-....A -..G...... -......T.. 
R10preT Q9   --------.. ...-G...A. .AGGGTTA.. ....-....A -..G...... -......T.. 
R11preT Q1   --------.. ..G-GCA... .-------.. .A..-A..A. -......... -......... 
R11preT Q2   --------.. ..G-GCA... .-------.. .A..-A..A. -......... -......... 
R11preT Q3   --------.. ..G-GCA... .-------.. .A..-A.... -......... -......... 
R11preT Q4   --------.. ..G-GCA... .-------.. .A..-A.... -......... -......... 
R11preT Q5   --------.. ..G-GCA... .-------.. .A..-...A. -......... -......... 
R11preT Q6   --------.. ..G-GCA... .-------.. .A..-...A. -......... -......... 
R11preT Q7   --------.. ..G-GCA... .-------.. .A..-..... -......... -......... 
R11preT Q8   --------.. ..G-GCA... .-------.. .A..-..... -......... -......... 
R11preT Q9   --------.. ..G-GCA... .-------.. ....-A..A. -......... -......... 
R11preT Q1   --------.. ..G-GCA... .-------.. ....-A..A. -......... -......... 
R11preT Q1   --------.. ..G-GCA... .-------.. ....-A.... -......... -......... 
R11preT Q1   --------.. ..G-GCA... .-------.. ....-A.... -......... -......... 
R11preT Q1   --------.. ..G-GCA... .-------.. ....-...A. -......... -......... 
R11preT Q1   --------.. ..G-GCA... .-------.. ....-...A. -......... -......... 
R11preT Q1   --------.. ..G-GCA... .-------.. ....-..... -......... -......... 
R11preT Q1   --------.. ..G-GCA... .-------.. ....-..... -......... -......... 
R12preT Q1   --------.. ...-A..... .-------.. ....-..... -......... -......... 
R12preT Q2   --------.. ...-A..... .-------.. ....-..... -......... -......... 
R12preT Q3   --------.. ...-G..... .-------.. ....-..... -......... -......... 
R12preT Q4   --------.. ...-G..... .-------.. ....-..... -......... -......... 
R13preT Q1   --------.. ..G-CCA.GC .-------.. ....-..... -......... -......... 
R13preT Q2   --------.. ..G-CCA.GC .-------.. ....-..... -..G...... -......... 
R15preT Q1   --------.. ...-G..... .-------.. G...-..... -..G...... -......-.. 
C1           --------.. ...-G..... .-------.. G.AG-A.... -..G...... -......... 
C2Q1         --------.. ..G-GCA... .-------.. ....-..... -....GA... -.G....... 
C2Q2         --------.. ..G-GCA... .-------.. ....-..... -....GA... -.G....... 
C3clone 1    --------.. ...-G..... .-------.. ....-..... -......... -......... 
C3clone 2    --------.. ...-G..... .-------.. ....-..... -......... -......... 
C4           --------.. ...GG-.... .-------.. ....-..... -......... -......... 
C6Q1         --------.. ...-G..... .-------.. ....-..... -..G...... -......-.. 
C6Q2         --------.. ...-G..... .-------.. ....-..... -..G...... -......... 
C7Q1         --------.. ...TG..... .-------.. ....-..... -......... -......... 
C7Q2         --------.. ...TG..... .-------.. ....-..... -......... -......... 
C7Q3         --------.. ...TG..... .-------.. ....-..... -......... -......... 
C7Q4         --------.. ...TG..... .-------.. ....-..... -......... -......... 
C8Q1         --------.. ...-G..... .-------.. ....-..... -......... -......... 
C8Q2         --------.. ...-G..... .-------.. ....-..... -......... -......... 
C8Q3         --------.. ...-G..... .-------.. ....-..... -......... -......... 
C8Q4         --------.. ...-G..... .-------.. ....-..... -......... -......... 
C9           --------.. ...-G..... .-------.. ....-..... -......... -......... 


             ....|....| ....|....| ....|....| ....|....| ....|....| ....|....| 
                      70         80         90        100        110        120             
AY624965     CC--TCCCGG GAGAGCCATA GTGGTCTGCG GAACCGGTGA GTACACCGGA ATCGCCGG-- 
AY624966     ..C-...... .......... .......... .......... .......... ........-- 
AY624967     ..C-...... .......... .......... .......... ..T....... ......A.-- 
AY624968     ..C-...... .......... .......... .......... ..T....... ........-- 
AY624969     ..C-...... .......... .......... .......... .......... ........-- 
AY624970     ..--...... .......... .......... .......... .......... ........-- 
AY624971     ..C-...... .......... .......... .......... .......... ........-- 
AY624972     ..C-...... .......... .......... .......... .......... ........-- 
AY624973     ..C-...... .......... .......... .......... .......... ........-- 
AY624974     ..--...... .......... .......... .......... .......... ........-- 
AY624975     ..--...... .......... .......... .......... .......... ........-- 
AY624976     ..C-...... .......... .......... .......... .......... ........-- 
AY624977     ..C-...... .......... .......... .......... .......... ........-- 
AY624978     ..C-...... .......... .......... .......... .......... ........-- 
AY624979     ..C-...... .......... .......... .......... .......... ........-- 
AY624980     ..C-...... .......... .......... .......... .......... ........-- 
AY624981     ..C-...... .......... .......... .......... .......... ........-- 
AY624982     ..C-...... .......... .......... .......... .......... ........-- 
AY624983     ..C-...... .......... .......... .......... .......... ........-- 
AY624984     ..--...... .......... .......... .......... .......... ........-- 
AY624985     ..C-...... .......... .......... .......... .......... ........-- 
AY624986     ..--...... .......... .......... .......... .......... ........-- 
NR7preT      ..C-...... .......... .......... .......... .......... ........-- 
NR7postT     ..C-...... .......... .......... .......... ..T....... ........-- 
BT1preT1     ..C-...... .......... .......... .......... .......... ........-- 
BT1preT2     ..C-...... .......... .......... .......... .......... ........-- 
BT1postT1    ..C-...... .......... .......... .......... .......... ........-- 
BT1postT2    ..C-...... .......... .......... .......... .......... ........-- 
BT1postT3    ..C-...... .......... .......... .......... .......... ........-- 
BT1postT4    ..C-...... .......... .......... .......... .......... ........-- 
BT1postT5    ..C-...... .......... .......... .......... .......... ........-- 
BT1postT6    ..C-...... .......... .......... .......... .......... ........-- 
BT2preT1     ..C-...... .......... .......... .......... .......... ........-- 
BT2preT2     ..C-...... .......... .......... .......... .......... ........-- 
BT2postT1    ..C-...... .......... .......... .......... .......... ........-- 
BT2postT2    ..C-...... .......... .......... .......... .......... ........-- 
BT2postT3    ..C-...... .......... .......... .......... .......... ........-- 
BT2postT4    ..C-...... .......... .......... .......... .......... ........-- 
BT3preT1     ..C-...... .......... .......... .......... .......... ........-- 
BT3preT2     ..C-...... .......... .......... .....T.... .......... ........-- 
BT3postT1    ..C-...... .......... .......... .......... .......... ........-- 
BT3postT2    ..C-...... .......... .......... .......... .......... ........-- 
BT3postT3    ..C-...... .......... .......... .....T.... .......... ........-- 
BT3postT4    ..C-...... .......... .......... .....T.... .......... ........-- 
BT3postT5    ..C-...... .......... .......... .......... .......... ........-- 
BT3postT6    ..C-...... .......... .......... .......... .......... ........-- 
BT3postT7    ..C-...... .......... .......... .....T.... .......... ........-- 
BT3postT8    ..C-...... .......... .......... .....T.... .......... ........-- 
BT4preT1     ..--...... .......... .......... .......... .......... ........-- 
BT4postT1    ..--...... .......... .......... .......... .......... ........-- 
BT4postT2    ..--...... .......... .......... .......... .......... ........-- 
BT4postT3    ..--...... .......... .....T.... .......... .......... ........-- 
BT4postT4    ..--...... .......... .....T.... .......... .......... ........-- 
BT5preT1     ..C-...... .......... .......... .......... .......... ........-- 
BT5preT2     ..C-...... .......... .......... .......... .......... ........-- 
BT5postT1    ..C-...... .......... .......... .......... .......... ........-- 
BT5postT2    ..C-...... .......... .......... .......... .......... ........-- 
BT5postT3    ..C-...... .......... .......... .......... .......... ........-- 
BT5postT4    ..C-...... .......... .......... .......... .......... ........-- 
BT5postT5    ..C-...... .......... .......... .......... .......... ........-- 
BT5postT6    ..C-...... .......... .......... .......... .......... ........-- 
BT5postT7    ..C-...... .......... .......... .......... .......... ........-- 
BT5postT8    ..C-...... .......... .......... .......... .......... ........-- 
R1preT Q1    ..C-...... .......... .......... .......... .......... ........-- 
R1preT Q2    ..C-...... .......... .......... .......... .......... ........-- 
R1preT Q3    ..C-...... .......... .......... .......... .......... ........-- 
R1preT Q4    ..C-...... .......... .......... .......... .......... ........-- 
R1preT Q5    ..C-...... .......... .......... .......... .......... ........-- 
R1preT Q6    ..C-...... .......... .......... .......... .......... ........-- 
R1preT Q7    ..C-...... .......... .......... .......... .......... ........-- 
R1preT Q8    ..C-...... .......... .......... .......... .......... ........-- 
R1preT Q9    ..C-...... .......... .......... .......... .......... ........-- 
R1preT Q10   ..C-...... .......... .......... .......... .......... ........-- 
R1preT Q11   ..C-...... .......... .......... .......... .......... ........-- 
R1preT Q12   ..C-...... .......... .......... .......... .......... ........-- 
R1preT Q13   ..C-...... .......... .......... .......... .......... ........-- 
R1preT Q14   ..C-...... .......... .......... .......... .......... ........-- 
R1preT Q15   ..C-...... .......... .......... .......... .......... ........-- 
R1preT Q16   ..C-...... .......... .......... .......... .......... ........-- 
R1preT Q17   ..C-...... .......... .......... .......... .......... ........-- 
R1preT Q18   ..C-...... .......... .......... .......... .......... ........-- 
R1preT Q19   ..C-...... .......... .......... .......... .......... ........-- 
R1preT Q20   ..C-...... .......... .......... .......... .......... ........-- 
R1preT Q21   ..C-...... .......... .......... .......... .......... ........-- 
R1preT Q22   ..C-...... .......... .......... .......... .......... ........-- 
R1preT Q23   ..C-...... .......... .......... .......... .......... ........-- 
R1preT Q24   ..C-...... .......... .......... .......... .......... ........-- 
R1preT Q25   ..C-...... .......... .......... .......... .......... ........-- 
R1preT Q26   ..C-...... .......... .......... .......... .......... ........-- 
R1preT Q27   ..C-...... .......... .......... .......... .......... ........-- 
R1preT Q28   ..C-...... .......... .......... .......... .......... ........-- 
R1preT Q29   ..C-...... .......... .......... .......... .......... ........-- 
R1preT Q30   ..C-...... .......... .......... .......... .......... ........-- 
R1preT Q31   ..C-...... .......... .......... .......... .......... ........-- 
R1preT Q32   ..C-...... .......... .......... .......... .......... ........-- 
R3preT Q1    ..C-...... .......... .......... .......... .......... ..T.....-- 
R3preT Q2    ..C-...... .......... .......... .......... .......... ........-- 
R4preT Q1    ..C-...... .......... .......... .......... .......... ........-G 
R4preT Q2    ..C-...... .......... .......... .......... .......... ......A.TG 
R5preT Q1    ..C-...... .......... .......... .......... .......... ........-- 
R6preT Q1    ..--...... .......... .......... .......... .......... ........-- 
R6preT Q2    ..--...... .......... .......... .......... .......... ........-- 
R8preT Q1    ..C-...... .......... .......... .......... .......... ........-- 
R8preT Q2    ..C-...... .......... .......... .......... .......... ........-- 
R9preT Q1    ..C-...... .......... .......... .......... .......... ........-- 
R9preT Q2    ..C-...... .......... .......... .......... .......... ........-- 
R10preT Q1   ..C-...... .......... .......... .......... .......... ........-- 
R10preT Q2   ..C-...... .......... .......... .......... .......... ........-- 
R10preT Q3   ..C-...... .......... .......... .......... .......... ........-- 
R10preT Q4   ..C-...... .......... .......... .......... .......... ........-- 
R10preT Q5   ..C-...... .......... .......... .......... .......... ........-- 
R10preT Q6   ..C-...... .......... .......... .......... .......... ........-- 
R10preT Q7   ..C-...... .......... .......... .......... .......... ........-- 
R10preT Q8   ..C-...... .......... .......... .......... .......... ........-- 
R10preT Q9   ..C-...... .......... .......... .......... .......... ........-- 
R11preT Q1   ..C-...... .......... .......... .......... .......... ........-- 
R11preT Q2   ..C-...... .......... .......... .......... .......... ........-- 
R11preT Q3   ..C-...... .......... .......... .......... .......... ........-- 
R11preT Q4   ..C-...... .......... .......... .......... .......... ........-- 
R11preT Q5   ..C-...... .......... .......... .......... .......... ........-- 
R11preT Q6   ..C-...... .......... .......... .......... .......... ........-- 
R11preT Q7   ..C-...... .......... .......... .......... .......... ........-- 
R11preT Q8   ..C-...... .......... .......... .......... .......... ........-- 
R11preT Q9   ..C-...... .......... .......... .......... .......... ........-- 
R11preT Q1   ..C-...... .......... .......... .......... .......... ........-- 
R11preT Q1   ..C-...... .......... .......... .......... .......... ........-- 
R11preT Q1   ..C-...... .......... .......... .......... .......... ........-- 
R11preT Q1   ..C-...... .......... .......... .......... .......... ........-- 
R11preT Q1   ..C-...... .......... .......... .......... .......... ........-- 
R11preT Q1   ..C-...... .......... .......... .......... .......... ........-- 
R11preT Q1   ..C-...... .......... .......... .......... .......... ........-- 
R12preT Q1   ..C-...... .......... .......... .......... .......... ........-- 
R12preT Q2   ..C-...... .......... .......... .......... .......... ........-- 
R12preT Q3   ..C-...... .......... .......... .......... .......... ........-- 
R12preT Q4   ..C-...... .......... .......... .......... .......... ........-- 
R13preT Q1   ..C-...... .......... .......... .......... .......... ........-- 
R13preT Q2   ..C-...... .......... .......... .......... .......... ........-- 
R15preT Q1   ..C-...... .......... .......... .......... .......... ........-- 
C1           ..C-...... .......... .......... .......... ..T....... ......A.-- 
C2Q1         ..T-C..--. .......... .......... .......... .......... ........-- 
C2Q2         ..T-C..--. .......... .......... .......... .......... ........-- 
C3clone 1    ..C-...... .......... .......... .......... .......... ........-- 
C3clone 2    ..C-...... .......... .......... .......... .......... ........-- 
C4           ..C-...... .......... .......... .......... ..T....... ........-- 
C6Q1         ..C-...... .......... .......... .......... .......... ........-- 
C6Q2         ..--...... .......... .......... .......... .......... ........-- 
C7Q1         ..C-...... .......... .......... .......... .......... ........-- 
C7Q2         ..C-...... .......... .......... .......... .......... ........-- 
C7Q3         ..C-...... .......... .......... .......... .......... ........-- 
C7Q4         ..C-...... .......... .......... .......... .......... ........-- 
C8Q1         ..--...... .......... .......... .......... .......... ........-- 
C8Q2         ..--...... .......... .......... .......... .......... ........-- 
C8Q3         ..--...... .......... .......... .......... .......... ........-- 
C8Q4         ..--...... .......... .......... .......... .......... ........-- 
C9           ..CC...... .......... .......... .......... .......... ........-- 


             ....|....| ....|....| ....|....| ....|....| ....|....| ....|....| 
                     130        140        150        160        170        180          
AY624965     GATGACCGGG TCCTTTCTTG GATCAA-CCC GCTCAATGCC CGGAAATTTG GGCGTGCCCC 
AY624966     .......... .......... ......-... .......... .......... .......... 
AY624967     ..C....... .......... ...TT.A... .......... T......... .......... 
AY624968     ..C....... .......... ..A...A... .......... ...C...... .......... 
AY624969     .......... .......... ......-... .......... .......... .......... 
AY624970     .......... .......... ...T..-... .......... .......... .......... 
AY624971     .......... .......... ......-... .......... .......... .......... 
AY624972     .......... .......... ...T..-... .......... .......... .......... 
AY624973     .......... .......... ...T..-... .......... .......... .......... 
AY624974     .......... .......... ...T..-... .......... .......... .......... 
AY624975     .......... .......... ...Y..-... .......... .......... .......... 
AY624976     .......... .......... ...T..-... .......... .......... .......... 
AY624977     .......... .......... ......-... .......... .......... .......... 
AY624978     .......... .......... ......-... .......... .......... .......... 
AY624979     .......... .......... ...T..-... .......... .......... .......... 
AY624980     .......... .......... ...T..-... .......... .......... .......... 
AY624981     .......... .......... ...T..-... .......... .......... .......... 
AY624982     .......... .......... ...T..-... .......... .......... .......... 
AY624983     .......... .......... ......-... .......... .......... .......... 
AY624984     ..Y....... .......... ..AT..-... .......... .......... .......... 
AY624985     .......... .......... ...T..-... .......... .......... .......... 
AY624986     .......... .......... ...T..A... .......... .......... .......... 
NR7preT      .......... .......... ...T..-... .......... .......... .......... 
NR7postT     ..C......- .......... ..A...A... .......... ...C...... .......... 
BT1preT1     .......... .......... ...T..-... .......... .......... .......... 
BT1preT2     .......... .......... ......-... .......... .......... .......... 
BT1postT1    .......... .......... ...T..-... .......... .......... .......... 
BT1postT2    .......... .......... ......-... .......... .......... .......... 
BT1postT3    .......... .......... ......-... .......... .......... .......... 
BT1postT4    .......... .......... ...T..-... .......... .......... .......... 
BT1postT5    .......... .......... ......-... .......... .......... .......... 
BT1postT6    .......... .......... ...T..-... .......... .......... .......... 
BT2preT1     .......... ...GGG.... ......-... .......... .......... .......... 
BT2preT2     .......... ...GGG.... ...T..-... .......... .......... .......... 
BT2postT1    .......... ...GGG.... ......-... .......... .......... .......... 
BT2postT2    .......... ...GGG.... ...T..-... .......... .......... .......... 
BT2postT3    .......... ...GGG.... ......-... .......... .......... .......... 
BT2postT4    .......... ...GGG.... ...T..-... .......... .......... .......... 
BT3preT1     .......... .......... ...T..-... .......... .......... .......... 
BT3preT2     .......... .......... ...T..-... .......... .......... .......... 
BT3postT1    .......... .......... ...T..-... .......... .......... .......... 
BT3postT2    .....T.... ........C. ...T..-... .......... .......... .......... 
BT3postT3    .......... .......... ...T..-... .......... .......... .......... 
BT3postT4    .....T.... ........C. ...T..-... .......... .......... .......... 
BT3postT5    .....T.... .......... ...T..-... .......... .......... .......... 
BT3postT6    .......... ........C. ...T..-... .......... .......... .......... 
BT3postT7    .......... ........C. ...T..-... .......... .......... .......... 
BT3postT8    .....T.... .......... ...T..-... .......... .......... .......... 
BT4preT1     .......... .......... ...T..-... .......... .......... .......... 
BT4postT1    .......... .......... ...T..-... .......... .......... .......... 
BT4postT2    .......... .......... ...T..-... .......... .......... .......... 
BT4postT3    .......... .......... ...T..-... .......... .......... .......... 
BT4postT4    .......... .......... ...T..-... .......... .......... .......... 
BT5preT1     .......... .......... ...T..-... .......... .......... .......... 
BT5preT2     .......... .......... ...T..-... .......... .......... .......... 
BT5postT1    .......... .......... ...T..-... .......... .......... .......... 
BT5postT2    .......... .......... ...T..-... .......... .......... .......... 
BT5postT3    .......... .......... ...T..-... .......... .......... ....A..... 
BT5postT4    .......... .......... ...T..-... .......... .......... ....A..... 
BT5postT5    .......... .......... ...T..-... .......... .......... ....A..... 
BT5postT6    .......... .......... ...T..-... .......... .......... ....A..... 
BT5postT7    .......... .......... ...T..-... .......... .......... .......... 
BT5postT8    .......... .......... ...T..-... .......... .......... .......... 
R1preT Q1    .......... .......... ...T..-... .......... .......... .......... 
R1preT Q2    .......... .......... ...T..-... .......... .......... .......... 
R1preT Q3    .......... .......... ...T..-... .......... .......... .......... 
R1preT Q4    .......... .......... ...T..-... .......... .......... .......... 
R1preT Q5    .......... .......... ...T..-... .......... .......... .......... 
R1preT Q6    .......... .......... ...T..-... .......... .......... .......... 
R1preT Q7    .......... .......... ...T..-... .......... .......... .......... 
R1preT Q8    .......... .......... ...T..-... .......... .......... .......... 
R1preT Q9    .......... .......... ...T..-... .......... .......... .......... 
R1preT Q10   .......... .......... ...T..-... .......... .......... .......... 
R1preT Q11   .......... .......... ...T..-... .......... .......... .......... 
R1preT Q12   .......... .......... ...T..-... .......... .......... .......... 
R1preT Q13   .......... .......... ...T..-... .......... .......... .......... 
R1preT Q14   .......... .......... ...T..-... .......... .......... .......... 
R1preT Q15   .......... .......... ...T..-... .......... .......... .......... 
R1preT Q16   .......... .......... ...T..-... .......... .......... .......... 
R1preT Q17   .......... .......... ...T..-... .......... .......... .......... 
R1preT Q18   .......... .......... ...T..-... .......... .......... .......... 
R1preT Q19   .......... .......... ...T..-... .......... .......... .......... 
R1preT Q20   .......... .......... ...T..-... .......... .......... .......... 
R1preT Q21   .......... .......... ...T..-... .......... .......... .......... 
R1preT Q22   .......... .......... ...T..-... .......... .......... .......... 
R1preT Q23   .......... .......... ...T..-... .......... .......... .......... 
R1preT Q24   .......... .......... ...T..-... .......... .......... .......... 
R1preT Q25   .......... .......... ...T..-... .......... .......... .......... 
R1preT Q26   .......... .......... ...T..-... .......... .......... .......... 
R1preT Q27   .......... .......... ...T..-... .......... .......... .......... 
R1preT Q28   .......... .......... ...T..-... .......... .......... .......... 
R1preT Q29   .......... .......... ...T..-... .......... .......... .......... 
R1preT Q30   .......... .......... ...T..-... .......... .......... .......... 
R1preT Q31   .......... .......... ...T..-... .......... .......... .......... 
R1preT Q32   .......... .......... ...T..-... .......... .......... .......... 
R3preT Q1    .......... .......... ......-... .......... .......... .......... 
R3preT Q2    .......... .......... ......-... .......... .......... .......... 
R4preT Q1    .......... .......... ......-... .......... .......... .......... 
R4preT Q2    .......... .......... ......-... .......... .......... .......... 
R5preT Q1    .......... .......... ...T..-... .......... .......... .......... 
R6preT Q1    .......... .......... ...T..-... .......... .......... .......... 
R6preT Q2    .......... .......... ...T..-... .......... .......... .......... 
R8preT Q1    .......... .......... ...T..-... .......... .......... .......... 
R8preT Q2    .......... .......... ...T..-... .......... .......... .......... 
R9preT Q1    .......... .......... ......-... .......... .......... .......... 
R9preT Q2    .......... .......... ...A..-... .......... .......... .......... 
R10preT Q1   .......... .......... ...T..-... .......... .......... .......... 
R10preT Q2   .......... .......... ...T..-... .......... .......... .......... 
R10preT Q3   .......... .......... ...T..-... .......... .......... .......... 
R10preT Q4   .......... .......... ...T..-... .......... .......... .......... 
R10preT Q5   .......... .......... ...T..-... .......... .......... .......... 
R10preT Q6   .......... .......... ...T..-... .......... .......... .......... 
R10preT Q7   .......... .......... ...T..-... .......... .......... .......... 
R10preT Q8   .......... .......... ...T..-... .......... .......... .......... 
R10preT Q9   .......... .......... ...T..-... .......... .......... .......... 
R11preT Q1   .......... .......... ...T..-... .......... .......... .......... 
R11preT Q2   .......... .......... ...T..-... .......... .......... .......... 
R11preT Q3   .......... .......... ...T..-... .......... .......... .......... 
R11preT Q4   .......... .......... ...T..-... .......... .......... .......... 
R11preT Q5   .......... .......... ...T..-... .......... .......... .......... 
R11preT Q6   .......... .......... ...T..-... .......... .......... .......... 
R11preT Q7   .......... .......... ...T..-... .......... .......... .......... 
R11preT Q8   .......... .......... ...T..-... .......... .......... .......... 
R11preT Q9   .......... .......... ...T..-... .......... .......... .......... 
R11preT Q1   .......... .......... ...T..-... .......... .......... .......... 
R11preT Q1   .......... .......... ...T..-... .......... .......... .......... 
R11preT Q1   .......... .......... ...T..-... .......... .......... .......... 
R11preT Q1   .......... .......... ...T..-... .......... .......... .......... 
R11preT Q1   .......... .......... ...T..-... .......... .......... .......... 
R11preT Q1   .......... .......... ...T..-... .......... .......... .......... 
R11preT Q1   .......... .......... ...T..-... .......... .......... .......... 
R12preT Q1   .......... .......... ...T..-... .......... .......... .......... 
R12preT Q2   .......... .......... ...T..-... .......... .......... .......... 
R12preT Q3   .......... .......... ...T..-... .......... .......... .......... 
R12preT Q4   .......... .......... ...T..-... .......... .......... .......... 
R13preT Q1   .......... .......... ...T..-... .......... .......... .......... 
R13preT Q2   .......... .......... ...T..-... .......... .......... .......... 
R15preT Q1   .......... .......... ...T..-... .......... .......... .......... 
C1           ..C....... .......... ...T..-... .G........ T......... .......... 
C2Q1         .......... .......... ......-... .......... .......... .......... 
C2Q2         .......... .......... ......-... .......... .......... .......... 
C3clone 1    .......... .......... ...T..-... .......... .......... .......... 
C3clone 2    .......... .......... ...T..-... .......... .......... .......... 
C4           ..C....... .......... ..A...A... .......... ...C...... .......... 
C6Q1         .......... .......... ...T..-... .......... .......... .......... 
C6Q2         .......... .......... ...T..-... .......... .......... ...A...... 
C7Q1         .......... .......... ...T..-... .......... .......... ..AA...... 
C7Q2         .......... .......... ...T..-... .......... .......... .CAA...... 
C7Q3         .......... .......... -..T..-... .......... .......... ..AAG..... 
C7Q4         .......... .......... ...T..-... .......... .......... .CAA...... 
C8Q1         .......... .......... ...T..-... .......... .......... .......... 
C8Q2         .......... .......... ......-... .......... .......... .......... 
C8Q3         .......... .......... ......-... .G........ .......... .......... 
C8Q4         .......... .......... ...T..-... .G........ .......... .......... 
C9           .......... .......... ...T..-... .......... .......... .C........ 


             ....|....| ....|....| ....|....| ....|....| ....|....| ....|....| 
                     190        200        210        220        230        240          
AY624965     -CGCAAGACT GCT---AGCC GAGTAGT--G TTGGGT-CGC GAAAGGCCTT GTGGTACTKC 
AY624966     -......... ...---.... .......--. ......-... ...G.C.TG. .KAC.G---- 
AY624967     -......... ...---.... ......C--. ......-T.. .......... .......MTG 
AY624968     -......... ...---.... .......--. ......-... .......... ......M.G. 
AY624969     -...G..... ...---.... .......--. ......-... .......... .......--- 
AY624970     -...G..... ...---.... .......--. ......-... ...-...... ........G- 
AY624971     -...G..... ...---.... .......--. ......-... .......... ......---- 
AY624972     -...G..... ...---.... .......--. ......-... .......... ........G- 
AY624973     -...G..... ...---.... .......--. ......-... .......T.. ....------ 
AY624974     -...G..... ...---.... .......--. ......-... .......... ........WG 
AY624975     -...G...S. ...---.... .......--. ......-... ...-...... ....------ 
AY624976     -...G..... ...---.... .......--. ......-... .......... .....CAAT- 
AY624977     -...G..... ...---.... .......--. ......-... .......... .......--- 
AY624978     -...G..... ...---.... .......--. ......-... .......... .......--- 
AY624979     -...G..... ...---.... .......--. ......-... .......... ......---- 
AY624980     --........ ...---.... .......--. ......-... .........G TG.TA.T--- 
AY624981     -...G..... ...---.... .......--. ......-... ..-------- ---------- 
AY624982     -...G..... ...---.... .......--. ......-... ...G.CGTG. ---------- 
AY624983     -...G..... ...---.... .......--. ......-... .......... .......--- 
AY624984     -...G..... ...---.... .......--. ......-... ---------- ---------- 
AY624985     -...G..... ...---.... .......--. ......-... .......... .--------- 
AY624986     -...G..... ...---.... .......--. ......-... ........C. TGT.GTACTG 
NR7preT      -...G..... ...---.... .......--. ......G... .......... ---------- 
NR7postT     -......... ...---.... .......--. ......G... .......... ---------- 
BT1preT1     -...G..... ...---.... .......--. ......-... .......... ---------- 
BT1preT2     -...G..... ...---.... .......--. ......-... .......... ---------- 
BT1postT1    -...G..... ...---.... .......--. ......-... .......... ---------- 
BT1postT2    -...G..... ...---.... .......--. ......-... .......... ---------- 
BT1postT3    -...G..... ...---.... .......--. ......-... .......... ---------- 
BT1postT4    -...G..... ...---.... .......--. ......-... .......... ---------- 
BT1postT5    -...G..... ...---.... .......--. ......-... .......... ---------- 
BT1postT6    -...G..... ...---.... .......--. ......-... .......... ---------- 
BT2preT1     -......... ...---.... .......--. ......-... .......... ---------- 
BT2preT2     -......... ...---.... .......--. ......-... .......... ---------- 
BT2postT1    -......... ...---.... .......--. ......-... .......... ---------- 
BT2postT2    -......... ...---.... ....C..--. ......-... .......... ---------- 
BT2postT3    -......... ...---.... ....C..--. ......-... .......... ---------- 
BT2postT4    -......... ...---.... .......--. ......-... .......... ---------- 
BT3preT1     -...G..... ...---.... .......--. ......-... .......... ---------- 
BT3preT2     -...G..... ...---.... .......--. ......-... .......... ---------- 
BT3postT1    -...G..... ...---.... .......--. ......-... .......... ---------- 
BT3postT2    -...G..... ...---.... .......--. ......-... .......... ---------- 
BT3postT3    -...G..... ...---.... .......--. ......-... .......... ---------- 
BT3postT4    -...G..... ...---.... .......--. ......-... .......... ---------- 
BT3postT5    -...G..... ...---.... .......--. ......-... .......... ---------- 
BT3postT6    -...G..... ...---.... .......--. ......-... .......... ---------- 
BT3postT7    -...G..... ...---.... .......--. ......-... .......... ---------- 
BT3postT8    -...G..... ...---.... .......--. ......-... .......... ---------- 
BT4preT1     -...G..... ...---..T. .......--. ......-... .......... ---------- 
BT4postT1    -...G..... ...---..T. .......--. ......-... .......... ---------- 
BT4postT2    -......... ...---..T. .......--. ......-... .......... ---------- 
BT4postT3    -...G..... ...---..T. .......--. ......-... .......... ---------- 
BT4postT4    -......... ...---..T. .......--. ......-... .......... ---------- 
BT5preT1     -......... ...---.... .......--. ....TCG... .......... ---------- 
BT5preT2     -......... ...---.... .......--. ....TCG... .......... ---------- 
BT5postT1    -......... ...---.... .......--. ....TCG... .......... ---------- 
BT5postT2    -......... ...---.... .......--. ....TCG... .......... ---------- 
BT5postT3    -......... ...---.... .......--. ....TCG... .......... ---------- 
BT5postT4    -......... ...---.... .......--C ....TCG... .......... ---------- 
BT5postT5    -......... ...---.... .......--. ....TCG... .......... ---------- 
BT5postT6    -......... ...---.... .......--C ....TCG... .......... ---------- 
BT5postT7    -......... ...---.... .......--C ....TCG... .......... ---------- 
BT5postT8    -......... ...---.... .......--C ....TCG... .......... ---------- 
R1preT Q1    -...G..... ...---.... .......--. ......-... .......... ---------- 
R1preT Q2    -...G..... ...---.... .......--. ......-... ..C....... ---------- 
R1preT Q3    -...G..... ...---.... .....T.--. ......-... .......... ---------- 
R1preT Q4    -...G..... ...---.... .....T.--. ......-... ..C....... ---------- 
R1preT Q5    -...G..... ...---.... .......--. ......-... .......... ---------- 
R1preT Q6    -...G..... ...---.... .......--. ......-... ..C....... ---------- 
R1preT Q7    -...G..... ...---.... .....T.--. ......-... .......... ---------- 
R1preT Q8    -...G..... ...---.... .....T.--. ......-... ..C....... ---------- 
R1preT Q9    -...G..... ...---.... .......--. ......-... .......... ---------- 
R1preT Q10   -...G..... ...---.... .....T.--. ......-... .......... ---------- 
R1preT Q11   -...G..... ...---.... .......--. ......-... ..C....... ---------- 
R1preT Q12   -...G..... ...---.... .....T.--. ......-... ..C....... ---------- 
R1preT Q13   -...G..... ...---.... .......--. ......-... .......... ---------- 
R1preT Q14   -...G..... ...---.... .......--. ......-... ..C....... ---------- 
R1preT Q15   -...G..... ...---.... .....T.--. ......-... .......... ---------- 
R1preT Q16   -...G..... ...---.... .....T.--. ......-... ..C....... ---------- 
R1preT Q17   -...G..... ...---.... .......--. ......-... .......... ---------- 
R1preT Q18   -...G..... ...---.... .......--. ......-... ..C....... ---------- 
R1preT Q19   -...G..... ...---.... .....T.--. ......-... .......... ---------- 
R1preT Q20   -...G..... ...---.... .....T.--. ......-... ..C....... ---------- 
R1preT Q21   -...G..... ...---.... .......--. ......-... .......... ---------- 
R1preT Q22   -...G..... ...---.... .......--. ......-... ..C....... ---------- 
R1preT Q23   -...G..... ...---.... .....T.--. ......-... .......... ---------- 
R1preT Q24   -...G..... ...---.... .....T.--. ......-... ..C....... ---------- 
R1preT Q25   -...G..... ...---.... .......--. ......-... .......... ---------- 
R1preT Q26   -...G..... ...---.... .....T.--. ......-... .......... ---------- 
R1preT Q27   -...G..... ...---.... .......--. ......-... ..C....... ---------- 
R1preT Q28   -...G..... ...---.... .....T.--. ......-... ..C....... ---------- 
R1preT Q29   -...G..... ...---.... .......--. ......-... .......... ---------- 
R1preT Q30   -...G..... ...---.... .......--. ......-... ..C....... ---------- 
R1preT Q31   -...G..... ...---.... .....T.--. ......-... .......... ---------- 
R1preT Q32   -...G..... ...---.... .....T.--. ......-... ..C....... ---------- 
R3preT Q1    -...G..... ...---.... .......--. ......-... .......... ---------- 
R3preT Q2    -...G..... ...---.... .......--. ......-... .......... ---------- 
R4preT Q1    -...G..... ...---.... .......--. ......-... .......... ---------- 
R4preT Q2    -...G..... ...---.... .......--. ......-... .......... ---------- 
R5preT Q1    -......... ...---.... .......--. ......-... .......... ---------- 
R6preT Q1    -......... ...---.... .......--. ......-... .......... ......T--- 
R6preT Q2    -......... ...---.... .......--. ......-... .......... .....CT--- 
R8preT Q1    -...G..... ...---.... .......--. ......-... .......... ---------- 
R8preT Q2    -...G..... ...---.... .......--. ......-... .......... ---------- 
R9preT Q1    -......... ...---.... .......--. ......-... .......... ---------- 
R9preT Q2    -......... ...---.... .......--. ......-... .......... ---------- 
R10preT Q1   -...G..... ...---.... .......--. ......-... .......... ---------- 
R10preT Q2   -...G..... ...---.... ....C..--. ......-... .......... ---------- 
R10preT Q3   -...G..... ...---.... ....T..--. ......-... .......... ---------- 
R10preT Q4   -...G..... ...---.... .......--. ......-... .......... ---------- 
R10preT Q5   -...G..... ...---.... ....C..--. ......-... .......... ---------- 
R10preT Q6   -...G..... ...---.... ....T..--. ......-... .......... ---------- 
R10preT Q7   -...G..... ...---.... .......--. ......-... .......... ---------- 
R10preT Q8   -...G..... ...---.... ....C..--. ......-... .......... ---------- 
R10preT Q9   -...G..... ...---.... ....T..--. ......-... .......... ---------- 
R11preT Q1   -...G..... ...---.... .......--. ......-... .......... ---------- 
R11preT Q2   -...G..... ...---.... .......--. ......-... ..C....... ---------- 
R11preT Q3   -...G..... ...---.... .......--. ......-... .......... ---------- 
R11preT Q4   -...G..... ...---.... .......--. ......-... ..C....... ---------- 
R11preT Q5   -...G..... ...---.... .......--. ......-... .......... ---------- 
R11preT Q6   -...G..... ...---.... .......--. ......-... ..C....... ---------- 
R11preT Q7   -...G..... ...---.... .......--. ......-... .......... ---------- 
R11preT Q8   -...G..... ...---.... .......--. ......-... ..C....... ---------- 
R11preT Q9   -...G..... ...---.... .......--. ......-... .......... ---------- 
R11preT Q1   -...G..... ...---.... .......--. ......-... ..C....... ---------- 
R11preT Q1   -...G..... ...---.... .......--. ......-... .......... ---------- 
R11preT Q1   -...G..... ...---.... .......--. ......-... ..C....... ---------- 
R11preT Q1   -...G..... ...---.... .......--. ......-... .......... ---------- 
R11preT Q1   -...G..... ...---.... .......--. ......-... ..C....... ---------- 
R11preT Q1   -...G..... ...---.... .......--. ......-... .......... ---------- 
R11preT Q1   -...G..... ...---.... .......--. ......-... ..C....... ---------- 
R12preT Q1   -......... ...---.... .......--. ......-... ....A.G.C. T--------- 
R12preT Q2   -......... ...---.... .......--. ......-... ......G.C. T--------- 
R12preT Q3   -......... ...---.... .......--. ......-... ....A.G.C. T--------- 
R12preT Q4   -......... ...---.... .......--. ......-... ......G.C. T--------- 
R13preT Q1   -...G..... ...---.... .......--. ......-... .......... ---------- 
R13preT Q2   -...G..... ...---.... .......--. ......-... .......... ---------- 
R15preT Q1   -...G..... ...---.... .......--. ......-... .......... ---------- 
C1           G..T.G.C.. ..C--A.... ..AG.ACCC. .....GGTTT CC........ ---------- 
C2Q1         --..G..... ..AGCC.... ....GT.--. --..T.-... .......... ---------- 
C2Q2         --..G..... ...GCC.... ....GT.--. --..T.-... .......... ---------- 
C3clone 1    -...G..... ...---.... .......--. ......-... .......... ---------- 
C3clone 2    -...G..... ...---.... .......--. ......-... .......... ---------- 
C4           -......... ...---.... .......--. ......-... .......... ---------- 
C6Q1         -...G..... ...---.... .......--. ......-... .......... ---------- 
C6Q2         -...G..... ...---.... .......--. ......-... .......... ---------- 
C7Q1         -......... ...---.... .......--. ......-... .......... ---------- 
C7Q2         -......... ...---.... .......--. ......-... .......... ---------- 
C7Q3         -......... ...---.... .......--. ......G... .......... ---------- 
C7Q4         -......... ...---.... .......--. ......-... .......... ---------- 
C8Q1         -...G..... ...---.... .......--. ......-... .......... ---------- 
C8Q2         -...G..... ...---.... .......--. ......G... .......... ---------- 
C8Q3         -...G..... ...---.... .......--. ......G... .......... ---------- 
C8Q4         -...G..... ...---.... .......--. ......G... .......... ---------- 
C9           -..T.G.C.. ..C--A.... ..AG.ACCC. .....GGTTT CC......C. ---------- 


             ....|..
                   
AY624965     R------
AY624966     -------
AY624967     CACTG--
AY624968     AYTGA--
AY624969     -------
AY624970     -------
AY624971     -------
AY624972     -------
AY624973     -------
AY624974     CCMTGAT
AY624975     -------
AY624976     -------
AY624977     -------
AY624978     -------
AY624979     -------
AY624980     -------
AY624981     -------
AY624982     -------
AY624983     -------
AY624984     -------
AY624985     -------
AY624986     -------
NR7preT      -------
NR7postT     -------
BT1preT1     -------
BT1preT2     -------
BT1postT1    -------
BT1postT2    -------
BT1postT3    -------
BT1postT4    -------
BT1postT5    -------
BT1postT6    -------
BT2preT1     -------
BT2preT2     -------
BT2postT1    -------
BT2postT2    -------
BT2postT3    -------
BT2postT4    -------
BT3preT1     -------
BT3preT2     -------
BT3postT1    -------
BT3postT2    -------
BT3postT3    -------
BT3postT4    -------
BT3postT5    -------
BT3postT6    -------
BT3postT7    -------
BT3postT8    -------
BT4preT1     -------
BT4postT1    -------
BT4postT2    -------
BT4postT3    -------
BT4postT4    -------
BT5preT1     -------
BT5preT2     -------
BT5postT1    -------
BT5postT2    -------
BT5postT3    -------
BT5postT4    -------
BT5postT5    -------
BT5postT6    -------
BT5postT7    -------
BT5postT8    -------
R1preT Q1    -------
R1preT Q2    -------
R1preT Q3    -------
R1preT Q4    -------
R1preT Q5    -------
R1preT Q6    -------
R1preT Q7    -------
R1preT Q8    -------
R1preT Q9    -------
R1preT Q10   -------
R1preT Q11   -------
R1preT Q12   -------
R1preT Q13   -------
R1preT Q14   -------
R1preT Q15   -------
R1preT Q16   -------
R1preT Q17   -------
R1preT Q18   -------
R1preT Q19   -------
R1preT Q20   -------
R1preT Q21   -------
R1preT Q22   -------
R1preT Q23   -------
R1preT Q24   -------
R1preT Q25   -------
R1preT Q26   -------
R1preT Q27   -------
R1preT Q28   -------
R1preT Q29   -------
R1preT Q30   -------
R1preT Q31   -------
R1preT Q32   -------
R3preT Q1    -------
R3preT Q2    -------
R4preT Q1    -------
R4preT Q2    -------
R5preT Q1    -------
R6preT Q1    -------
R6preT Q2    -------
R8preT Q1    -------
R8preT Q2    -------
R9preT Q1    -------
R9preT Q2    -------
R10preT Q1   -------
R10preT Q2   -------
R10preT Q3   -------
R10preT Q4   -------
R10preT Q5   -------
R10preT Q6   -------
R10preT Q7   -------
R10preT Q8   -------
R10preT Q9   -------
R11preT Q1   -------
R11preT Q2   -------
R11preT Q3   -------
R11preT Q4   -------
R11preT Q5   -------
R11preT Q6   -------
R11preT Q7   -------
R11preT Q8   -------
R11preT Q9   -------
R11preT Q1   -------
R11preT Q1   -------
R11preT Q1   -------
R11preT Q1   -------
R11preT Q1   -------
R11preT Q1   -------
R11preT Q1   -------
R12preT Q1   -------
R12preT Q2   -------
R12preT Q3   -------
R12preT Q4   -------
R13preT Q1   -------
R13preT Q2   -------
R15preT Q1   -------
C1           -------
C2Q1         -------
C2Q2         -------
C3clone 1    -------
C3clone 2    -------
C4           -------
C6Q1         -------
C6Q2         -------
C7Q1         -------
C7Q2         -------
C7Q3         -------
C7Q4         -------
C8Q1         -------
C8Q2         -------
C8Q3         -------
C8Q4         -------
C9           -------
